# The Formation of Cr-Al Spinel under a Reductive Atmosphere

**DOI:** 10.3390/ma14123218

**Published:** 2021-06-10

**Authors:** Oleksandr Shtyka, Waldemar Maniukiewicz, Radoslaw Ciesielski, Adam Kedziora, Viktar Shatsila, Tomasz Sierański, Tomasz Maniecki

**Affiliations:** Institute of General and Ecological Chemistry, Lodz University of Technology, 90924 Lodz, Poland; waldemar.maniukiewicz@p.lodz.pl (W.M.); radoslaw.ciesielski@p.lodz.pl (R.C.); adam.kedziora@p.lodz.pl (A.K.); viktar.shatsila@dokt.p.lodz.pl (V.S.); tomasz.sieranski@p.lodz.pl (T.S.); tomasz.maniecki@p.lodz.pl (T.M.)

**Keywords:** Al_2_O_3_, Cr_2_O_3_, Cr, CrAlO_3_, spinel, reduction process

## Abstract

In the present work, for the first time, the possibility of formation of CrAl_2_O_4_ was shown from the equimolar mixture of co-precipitated Al_2_O_3_ and Cr_2_O_3_ oxides under a reductive environment. The crystallographic properties of the formed compound were calculated using the DICVOL procedure. It was determined that it has a cubic crystal structure with space group Fd-3m and a unit cell parameter equal to 8.22(3) Å. The formed CrAl_2_O_4_ is not stable under ambient conditions and easily undergoes oxidation to α-Al_2_O_3_ and α-Cr_2_O_3_. The overall sequence of the phase transformations of co-precipitated oxides leading to the formation of spinel structure is proposed.

## 1. Introduction

Aluminum oxide is an important inorganic compound that is extensively used in many industrial applications. It is also an important mineral (e.g., corundum) and a precious gemstone (e.g., ruby and sapphire). It has many crystalline polymorphic phases such as α-Al_2_O_3_, γ-Al_2_O_3_, δ-Al_2_O_3_, θ-Al_2_O_3_, η-Al_2_O_3_, κ-Al_2_O_3_, χ-Al_2_O_3_, and ρ-Al_2_O_3_ [1]. Among them, α-Al_2_O_3_ is the most stable alumina phase. The corundum structure consists essentially of a dense arrangement of oxygen ions in hexagonal closest packing with Al^3+^ ions occupying two-thirds of the available octahedrally coordinated sites.

Due to its structure, α-Al_2_O_3_ has some valuable physical and chemical properties such as good acid, alkali, and heat resistances; and high hardness and strength. It is widely used in different fields such as ceramics, surface protective layer materials, refractory materials, and optical materials [2].

Another commonly used form of alumina is γ-Al_2_O_3_. Its structure can be regarded as spinel type in which oxygen atoms are arranged in a cubic close packing and Al atoms occupy the octahedrally and tetrahedrally coordinated sites. γ-Al_2_O_3_, which is also called activated alumina, has a large surface area and strong adsorption capacity, and therefore is widely employed as absorbents, catalyst supports, chromatographic media, and ion exchanges [3,4,5].

Aluminum oxides are usually obtained via the heat treatment of aluminum hydroxides, e.g., boehmite or aluminum salts. The formation of one or another form of alumina depends mainly on the type and properties of the starting material and conditions of the process. In general, all of the transition aluminas transfer into the other modifications at about 600 °C and all are transformed into the thermally stable α-Al_2_O_3_ at temperatures above 1000 °C [6].

The physicochemical properties of Al_2_O_3_ can be considerably altered by incorporation of second phase particles such as ZrO_2_, SiC, SnO_2_, B_4_C, TiC, Cr_3_C_2_, and Cr_2_O_3_ [7,8,9,10]. Such modifications were reported to be beneficial for improving the mechanical properties of Al_2_O_3_-based ceramics [11,12]. Moreover, it was reported that the catalysts supported on Al_2_O_3_-based binary systems are superior in comparison with those supported on Al_2_O_3_ alone [13].

An interesting approach to modifying the properties of Al_2_O_3_ is the incorporation of Cr_2_O_3_ into its structure. Corundum (Al_2_O_3_) and eskolaite (Cr_2_O_3_) are isostructural, and Al^3+^ and Cr^3+^ ions have similar ionic radii. For this reason, they have similar unit cell parameters with a mismatch of ~4% [14]. Therefore, at high temperatures, Cr_2_O_3_ may form a substitutional solid solution in an Al_2_O_3_ lattice by exchange with Al^3+^ ions over the full range of compositions without formation of any eutectic. Depending on the content of Cr_2_O_3_ or Al_2_O_3_, these solutions can be divided into corundum and eskolaite by the dominant-constituent rule [15]. These materials are used as refractory materials as well as in several other applications [16].

The process of formation of substitutional solid solutions is energy-intensive and requires high temperatures at which the formation of spinel structure compounds can also occur. For instance, Sako [17] and Ping [18] reported the formation of MgAl_2_O_3_ from the corresponding oxides at temperatures as high as 1000 °C. The ordinary spinel structure, with a general formula of AB_2_O_4_ (where A^2+^ and B^3+^ are usually metal ions), is related to the arrangement of the octahedra that are the main framework of the spinel structure. This can explain relatively high hardness and high density usually observed for this group of compounds. In the normal spinel structure, the O^2-^ ions form a face-centered cubic array with A^2+^ and B^3+^ cations occupying one-eighth of the tetrahedrally and one-half of the octahedrally coordinated sites, respectively. In an inverse spinel B(AB)O_4_, alternatively, one-eighth of the tetrahedrally coordinated sites are occupied by cation B^3+^ whereas A^2+^ and B^3+^ each occupy one-quarter of the octahedrally coordinated sites. Usually, spinel compounds crystallize in the high symmetry space group type *Fd-3m*, but lower symmetries also occur [19]. The mechanism of formation of spinel compounds was investigated by many researchers, particularly Rossi [20], Carter [21], and Navias [22], who performed mechanistic studies related to the formation of magnesium aluminate spinel. Their results showed that the reaction proceeds through interdiffusion of Al^3+^ and Mg^2+^ ions through the oxygen array. Particularly, Al^3+^ ions migrate from the alumina particles to the magnesia ones and Mg^2+^ diffuses in the opposite direction, leading to the spinel layer formation at the interface between the alumina and magnesia particles.

The aim of the current research work was to investigate the possibility of the formation of a spinel structure from the aluminum and chromium oxides. It was assumed that under conditions of high temperature and a reductive atmosphere the formation of Cr^2+^ ions may occur, which would further diffuse into an Al_2_O_3_ matrix.

Moreover, we attempted to investigate the phase transformation of both aluminum and chromium oxides and the interaction between them as a function of temperature.

## 2. Experimental

### 2.1. Preparation of Samples

The mixture of aluminum and chromium oxides was prepared by a co-precipitation method using water solutions of corresponding nitrates as precursors and ammonia water as the precipitator. A mixed metal nitrate solution was prepared with a total concentration of metals equal to 1 M. The nominal Cr/Al molar ratio was 1:1. The ammonia was added into the metal solution until pH = 7.5 under vigorous stirring and slight heating (50 °C). The reaction was carried out for 24 h. After reaction, precipitates were filtered, washed with distilled water, dried in a vacuum dryer below 0.10 atm at 200 °C for 16 h, and then calcined in air at 400 °C for 4 h. Prior to the XRD measurements, the samples were reduced in a stream of pure hydrogen at temperatures ranging from 400 to 1450 °C overnight. Then, the obtained samples were cooled in the flow of an inert gas to ambient temperature and sealed to avoid contact with the air.

### 2.2. Physicochemical Characterization

The phase transformation of the prepared mixture as a function of temperature was investigated by X-ray diffraction analysis. The diffraction patterns were collected using a PANalytical X’Pert Pro MPD diffractometer in Bragg Brentano reflection geometry (Malvern Panalytical Ltd., Royston, UK). The diffractometer was equipped with a CuK_α_ radiation source (λ = 1.5418 Å). Data were collected in the 2θ range of 5°–80° with a step size of 0.0167° and exposure per step of 27 s.

For qualitative and Rietveld quantitative phase analyses, the PANalytical High Score software package (ver. 4.9) was used, combined with the International Centre for Diffraction Data’s (ICDD) powder diffraction file (PDF-2 ver. 2020) database of standard reference materials. The structural details for Rietveld quantitative analyses were obtained from the Crystallography Open Database [23]. To estimate the full width at half maximum (FWHM) of the diffraction profiles, the pseudo-Voigt function was applied. Then, after taking into account the broadening caused by the diffractometer, the FWHM was used to calculate the size of the crystallites using the Scherrer equation.

(1) $$D_{hkl}=\frac{K\lambda }{\beta_{D}\cos\left(\theta_{max}\right)}$$

where K = 0.9, a dimensionless shape factor; λ is the wavelength of scattered X-ray radiation; *β_D_* is the full width at half maximum of the *hkl* diffraction peak; and *θ_max_* is the scattering angle at the maximum of the hkl diffraction peak. The unit cell parameters for CrAl_2_O_4_ were determined by the DICVOL04 algorithm [24].

### 2.3. Theoretical Calculations

In order to confirm the conjecture formulated on the basis of the experimental data, the proposed spinel structure of CrAl_2_O_4_ was optimized by computing the analytical gradients of the energy with respect to both unit cell parameters and atom coordinates, treating CrAl_2_O_4_ as a 3-dimensional crystal system. The calculations were performed using CRYSTAL17 [25,26] and we employed B3LYP density functional with D3 version of Grimme’s dispersion [27]. The used basis set was the POB double-ζ polarization basis set (POB-DZVP) [28]. The initial structural parameters, which were taken as an input, were those found for the spinel structure of MgAl_2_O_4_ [29]. The data from the calculations were then compared with the experimental ones.

## 3. Results and Discussion

The formation of either separate aluminum and chromium oxides or their substitutional solution during the dehydration of corresponding hydroxides is governed mainly by the temperature of the process and the molar ratio of aluminum to chromium. According to the literature, at a temperature as high as 1250 °C, these oxides can form Cr_x_Al_2−x_O_3_ solid solution over a broad range of compositions (0 ≤ x ≤ 2). However, at a lower temperature, a miscibility gap is present, which originates from the different mechanisms of dehydration of aluminum and chromium hydroxides [30,31]. For instance, the dehydration of chromium hydroxide leads directly to the formation of α-Cr_2_O_3_ at a temperature above 400 °C, whereas the dehydration of aluminum hydroxide proceeds through the formation of aluminum oxyhydroxides, and then via different metastable alumina polymorphs, such as γ, δ, and θ, whose structure differs significantly from α-Cr_2_O_3_. The formation of α-Al_2_O_3_, which is isostructural with eskolaite, occurs only at a temperature above 1000 °C [32].

**Figure 1:**
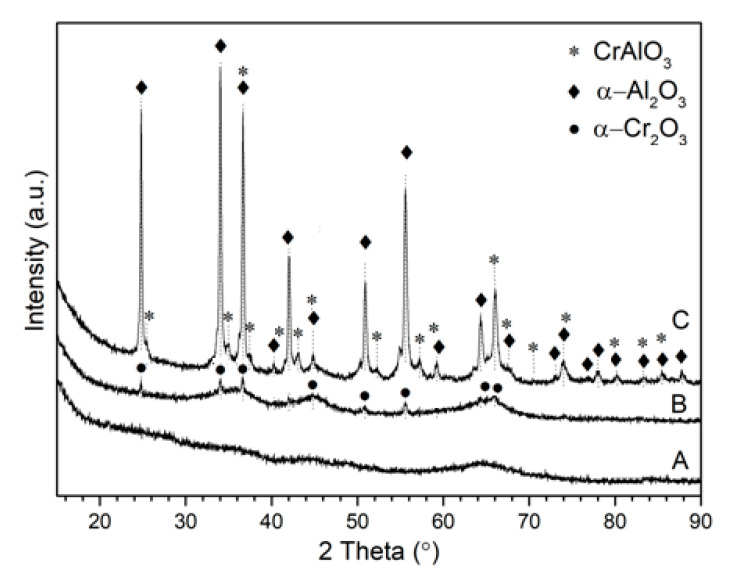
XRD patterns of the sample after calcination at 400 °C (**A**) and after subsequent reduction at 600 °C (**B**) and 800 °C (**C**).

The XRD measurements of the sample after calcination at 400 °C revealed that the formed oxides are amorphous, as indicated by the absence of any diffraction peaks (Figure 1). At a higher reduction temperature (600 °C), the weak broad diffraction maxima appeared at 2θ ca. 24.74°, 34.04°, 36.57°, and 55.53°, characteristic of the α-Cr_2_O_3_ phase (JCPDS Card No. 01-082-3794).

This indicates that at this temperature, only chromium oxide started to crystallize, which is consistent with data reported in the literature [14]. The further increase in temperature up to 800 °C resulted in an intensification and sharpening of those maxima as well as the appearance of new small diffraction peaks related to α-Al_2_O_3_ phase (JCPDS Card No. 01-088-0826). Moreover, diffraction peaks of α-Cr_2_O_3_ were slightly shifted toward a higher angle, indicating the formation of (Al,Cr)_2_O_3_ substitution solution. The unit cell parameters of the formed solid solution calculated from the peak (012) offset is presented in Table 1.

Based on a comparison with literature data [31], the content of Cr_2_O_3_ in solid solution was found to be ca. 80%. The formation of (Al,Cr)_2_O_3_ solid solution did not exclude the presence of isolated α-Cr_2_O_3_ phase, which is not detectable due to the overlap of diffraction peaks.

**Figure 2:**
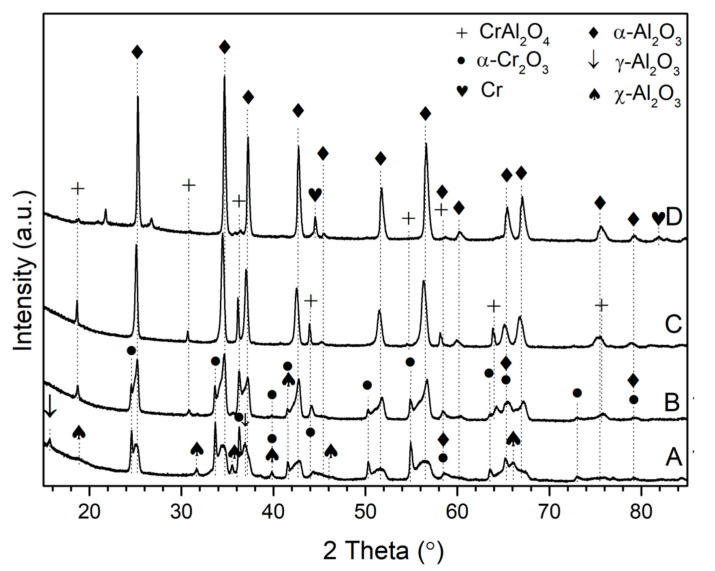
XRD patterns of the sample after reduction at 1000 (**A**), 1150 (**B**), 1300 (**C**), and 1450 (**D**) °C.

**Figure 3:**
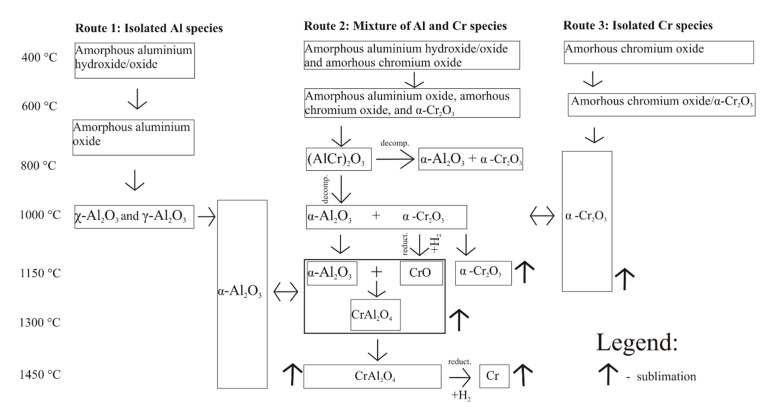
Phase transformation of co-precipitated Al and Cr oxides as a function of temperature.

Notably, no other crystallographic phases of alumina were observed at this temperature. On the other hand, when the reduction process was performed at 1000 °C, the diffraction maxima of metastable alumina polymorphs appeared. Particularly, the characteristic diffraction peaks of γ-Al_2_O_3_ (JCPDS Card No. 01-074-4629) and χ-Al_2_O_3_ (JCPDS Card No. 00-051-0769) phases were observed at 31.66°, 36.91°, and 45.12°; and at 7.76°, 15.52°, and 33.42°, respectively (Figure 2). Such results imply that the formation of α-Al_2_O_3_ grains proceeds via two different pathways, depending on the initial location of their formerly amorphous form (Figure 3). The Al_2_O_3_ located in close proximity to chromium oxide tends to nucleate directly on its surface and then grow as α-Al_2_O_3_ (Figure 3, Route 2)_._ The process can proceed at temperatures as low as 450 °C. On the contrary, the isolated grains of aluminum oxide crystallize as metastable forms, which then undergo structural transformation to thermodynamically stable α-Al_2_O_3_ (Figure 3, Route 1). The process is usually accomplished at temperatures above 1000 °C. The presence of both χ- and γ- may indicate that the dehydration process proceeds from different alumina precursors. Particularly, it was reported that the γ is the first Al_2_O_3_ polymorph that is formed during the calcination/dehydration of boehmite, whereas χ-Al_2_O_3_ is initially formed from the gibbsite. Both hydroxides can be originally formed during the precipitation stage and/or as a result of decomposition of gibbsite to boehmite during calcination process. The formed (Al,Cr)_2_O_3_ solution was not stable and decomposed under the investigated conditions, as evidenced by the disappearance of the corresponding diffraction maxima.

At 1150 °C, the XRD measurements revealed the presence of diffraction peaks at 2θ ca. 18.68°, 30.79°, 36.29°, 44.35°, and 64.68°. A good matching of these peaks to the MgAl_2_O_4_ standard was noted (JCPDS Card No. 01-075-0713). In order to confirm the experimental data, theoretical calculations were performed. Assuming isostructurality with MgAl_2_O_4_, good agreement between the experimental data and theoretical calculations was noted. On this basis, we think that a CrAl_2_O_4_ compound with a spinel structure can be formed. The ideal unit cell of the CrAl_2_O_4_ can be expressed as Cr_8_Al_16_O_32_, in which 32 oxygen anions (occupying 48f positions), are face-centered cubic close packed with a space group Fd-3m, with eight CrAl_2_O_4_ units per cubic cell. The Cr^2+^ ions occupy tetrahedral 8a symmetry position between O^2−^ ions and the Al^3+^ ions are sited in octahedral 16d sites. To confirm the CrAl_2_O_4_ structure, we calculated the bond-valence parameters, as proposed by Gagné and Hawthorne [33], at the tetrahedrally and octahedrally coordinated sites. The bond valence sum at the four-fold site is 2.26 v.u., which is a little larger than the +2 expected for the Cr^2+^ cation; the bond valence sum at the six-fold site is 2.92 v.u., which is a little smaller than the +3 expected for the Al^3+^ cation. These bond valence values not only confirm that Cr^2+^ and Al^3+^ are the dominant cations at the tetrahedrally and octahedrally coordinated sites, respectively, but they also indicate the occurrence of a partial Cr-Al disorder (likely less than 10% in terms of Cr^2+^ content) over the tetrahedrally and octahedrally coordinated sites. This type of disorder is always present in Cr-bearing MgAl_2_O_4_ spinels [34]. Cámara et al. [35] reported occurrence of the mineral dellagiustaite, with ideal formula V^2+^Al_2_O_4_, formed under super-reduced geological conditions from high-temperature melts trapped in corundum aggregates. Since dellagiustaite is an inverse spinel, the V^2+^ cations prefer six-fold coordination, unlike Cr^2+^ cations, which prefer four-fold coordination in our spinel structure. In our opinion, the spinel CrAl_2_O_4_ may be found in nature under very reducing conditions typical of basaltic systems [36]. However, no minerals containing Cr^2+^ have been reported so far [37]. In conclusion, to the best of our knowledge, the CrAl_2_O_4_ compound has not been described in the literature yet. Table 2 presents the comparison of the unit cell parameters and crystallographic data for the newly formed compound, calculated using the DICVOL04 procedure and the CRYSTAL17 package.

On the basis of the obtained results, it can be postulated that at temperatures above 1150 °C, the α-Cr_2_O_3_ undergoes reduction to CrO, the chromium ions of which further diffuse into an α-Al_2_O_3_ matrix, forming CrAl_2_O_4_. At further increasing temperatures, the agglomeration of the formed compounds was observed as evidenced by the increase in crystallite size of both spinel compound and alumina. Particularly, the size of the α-Al_2_O_3_ particle increased from 29 nm at 1150 °C to 54 nm at 1450 °C, whereas the size of the crystallites of CrAl_2_O_4_ increased from 27 nm at 1150 °C to 125 nm at 1300 °C. In general, the increase in temperature beyond 1000 °C resulted in a gradual decrease in chromium-containing compounds due to their sublimation (Table 3).

At 1450 °C, small residual amounts of metallic chromium were observed, which formed as a result of a reduction of the spinel compound. All attempts to obtain pure CrAl_2_O_4_ spinel in order to study its physical properties more closely have failed. When exposed to ambient conditions, the Cr-Al spinel structure is metastable and readily undergoes oxidation to individual oxides. The general scheme of phase transformation of co-precipitated oxides as a function of temperature is depicted in Figure 3.

## 4. Summary

The obtained results showed that the crystallization of atomically mixed amorphous Al–Cr oxides proceeds first via the formation of α-Cr_2_O_3_, followed by the nucleation and growth of α-Al_2_O_3_ on its surface. Isolated grains of Al_2_O_3_ are also present as indicated by the crystallization of metastable alumina phases, which are subsequently converted into α-Al_2_O_3_ at high temperature. Under harsh reductive conditions, the investigated oxides are partially transformed to Cr–Al spinel compound. It is proposed that the formation of this structure is due to the formation of Cr^2+^ ions (because of reduction of α-Cr_2_O_3_) and then interdiffusion of Al^3+^ and Cr^2+^ ions through the oxygen array.

## Figures and Tables

**Table 1:** The unit cell parameters of (Al,Cr)_2_O_3_ and individual oxides.

Peak (012)	2θ (°)	a (Å)	c (Å)
Cr_2_O_3_	24.732	4.960 (1)	13.598 (4)
(Al,Cr)_2_O_3_ (~80% Cr_2_O_3_)	24.786	4.900 (1)	13.435 (3)
α-Al_2_O_3_	25.566	4.760 (1)	12.997 (3)

**Table 2:** Crystallographic analysis of CrAl_2_O_4_ (isostructural with MgAl_2_O_4_).

Crystal Data					
Experimental			Theoretical		
Crystal System	Space Group	a (Å)	Crystal System	Space Group	a (Å)
Cubic	Fd-3m	8.22 (3)	Cubic	Fd-3m	8.106 (2)
(hkl)	*d_hkl_*		(hkl)	*d_hkl_*	
(111)	4.75		(111)	4.680	
(022)	2.91		(022)	2.866	
(131)	2.48		(131)	2.444	
-	-		(222)	2.340	
(040)	2.16		(040)	2.206	
-	-		(133)	1.860	
-	-		(242)	1.655	
-	-		(151)	1.560	
(044)	1.45		(044)	1.433	
-	-		(153)	1.370	
-	-		(244)	1.351	

Please find the CIF and JPG file in Supplementary Materials.

**Table 3:** Phase composition of the investigated samples during reduction at different temperatures.

Temperature, °C	Phase Composition	Crystallite Size, nm *	Relative Concentration, %
600	α-Cr_2_O_3_	12	100
800	α-Al_2_O_3_	~5	28
	CrAlO_3_	50	72
1000	α-Al_2_O_3_	15	45
	χ-Al_2_O_3_	11	7
	γ-Al_2_O_3_	19	5
	α-Cr_2_O_3_	49	43
1150	α-Al_2_O_3_	29	55
	α-Cr_2_O_3_	20	21
	CrAl_2_O_4_	27	24
1300	α-Al_2_O_3_	40	85
	CrAl_2_O_4_	125	15
1450	α-Al_2_O_3_	54	94
	CrAl_2_O_4_	25	3
	Cr	60	3

* The error in estimating the crystallite size is about 1–2 nm.

## Data Availability

The data presented in this study are available on request from the corresponding author.

## Supplementary Materials

The following are available online at https://www.mdpi.com/article/10.3390/ma14123218/s1. CIF and JPG file of the CrAl_2_O_4_ compound.

## References

[1] Shirai T., Watanabe H., Fuji M., Takahashi M. (2009). Structural Properties and Surface Characteristics on Aluminum Oxide Powders. Annu. Rep. Adv. Ceram. Res. Cent. Nagoya Inst. Technol..

[2] Abyzov A.M. (2019). Aluminum Oxide and Alumina Ceramics (review). Part 1. Properties of Al_2_O_3_ and Commercial Production of Dispersed Al_2_O_3_. Refract. Ind. Ceram..

[3] Trueba M., Trasatti S.P. (2005). γ-alumina as a support for catalysts: A review of fundamental aspects. Eur. J. Inorg. Chem..

[4] Chu T.P.M., Nguyen N.T., Vu T.L., Dao T.H., Dinh L.C., Nguyen H.L., Hoang T.H., Le T.S., Pham T.D. (2019). Synthesis, characterization, and modification of alumina nanoparticles for cationic dye removal. Materials.

[5] Wang Z., Wu W., Bian X., Wu Y. (2016). Synthesis and characterization of amorphous Al_2_O_3_ and γ-Al_2_O_3_ by spray pyrolysis. Green Process. Synth..

[6] Digne M., Sautet P., Raybaud P., Toulhoat H., Artacho E. (2002). Structure and stability of aluminum hydroxides: A theoretical study. J. Phys. Chem. B.

[7] Karabulut Ş., Gökmen U., Çinici H. (2016). Study on the mechanical and drilling properties of AA7039 composites reinforced with Al_2_O_3_/B_4_C/SiC particles. Compos. Part B Eng..

[8] Yang K., Zhou X., Zhao H., Tao S. (2011). Microstructure and mechanical properties of Al_2_O_3_–Cr_2_O_3_ composite coatings produced by atmospheric plasma spraying. Surf. Coat. Technol..

[9] Cai K.F., McLachlan D.S., Axen N., Manyatsa R. (2002). Preparation, microstructures and properties of Al_2_O_3_-TiC composites. Ceram. Int..

[10] Tuan W.H., Chen R.Z., Wang T.C., Cheng C.H., Kuo P.S. (2002). Mechanical properties of Al_2_O_3_/ZrO_2_ composites. J. Eur. Ceram. Soc..

[11] Hirata T., Morimoto T., Deguchi A., Uchida N. (2002). Corrosion resistance of alumina-chromia ceramic materials against molten slag. Mater. Trans..

[12] Kafkaslıoğlu Yıldız B., Yılmaz H., Tür Y.K. (2019). Evaluation of mechanical properties of Al_2_O_3_–Cr_2_O_3_ ceramic system prepared in different Cr_2_O_3_ ratios for ceramic armour components. Ceram. Int..

[13] Kim K.J., Chang C.H., Ahn H.G. (2015). The effect of zinc oxide addition to alumina-supported gold catalyst in low temperature carbon monoxide oxidation. J. Nanosci. Nanotechnol..

[14] Eklund P., Sridharan M., Sillassen M., Bøttiger J. (2008). α-Cr_2_O_3_ template-texture effect on α-Al_2_O_3_ thin-film growth. Thin Solid Films.

[15] Bosi F., Hatert F., Hålenius U., Pasero M., Miyawaki R., Mills S.J. (2019). On the application of the IMA−CNMNC dominant-valency rule to complex mineral compositions. Mineral. Mag..

[16] Zhao P., Zhao H., Yu J., Zhang H., Gao H., Chen Q. (2018). Crystal structure and properties of Al_2_O_3_–Cr_2_O_3_ solid solutions with different Cr_2_O_3_ contents. Ceram. Int..

[17] Sako E.Y., Braulio M.A.L., Zinngrebe E., Van Der Laan S.R., Pandolfelli V.C. (2012). Fundamentals and applications on in situ spinel formation mechanisms in Al_2_O_3_-MgO refractory castables. Ceram. Int..

[18] Ping L.R., Azad A.M., Dung T.W. (2001). Magnesium aluminate (MgAl_2_O_4_) spinel produced via self-heat-sustained (SHS) technique. Mater. Res. Bull..

[19] Bosi F., Biagioni C., Pasero M. (2019). Nomenclature and classification of the spinel supergroup. Eur. J. Mineral..

[20] Rossi R.C., Fulrath R.M. (1963). Epitaxial Growth of Spinel by Reaction in the Solid State. J. Am. Ceram. Soc..

[21] CARTER R.E. (1961). Mechanism of Solid-state Reaction Between Magnesium Oxide and Aluminum Oxide and Between Magnesium Oxide and Ferric Oxide. J. Am. Ceram. Soc..

[22] NAVIAS L. (1961). Preparation and Properties of Spinel Made by Vapor Transport and Diffusion in the System MgO-Al_2_O_3_. J. Am. Ceram. Soc..

[23] Quirós M., Gražulis S., Girdzijauskaitė S., Merkys A., Vaitkus A. (2018). Using SMILES strings for the description of chemical connectivity in the Crystallography Open Database. J. Cheminform..

[24] Boultif A., Loueer D. (1991). Indexing of powder diffraction patterns for low-symmetry lattices by the successive dichotomy method. J. Appl. Crystallogr..

[25] Dovesi R., Erba A., Orlando R., Zicovich-Wilson C.M., Civalleri B., Maschio L., Rérat M., Casassa S., Baima J., Salustro S. (2018). Quantum-mechanical condensed matter simulations with CRYSTAL. Wiley Interdiscip. Rev. Comput. Mol. Sci..

[26] Dovesi R., Saunders V.R., Roetti C., Orlando R., Zicovich-Wilson C.M., Pascale F., Civalleri B., Doll K., Harrison N.M., Bush I.J. (2016). CRYSTAL 14: User’s Manual.

[27] Grimme S., Antony J., Ehrlich S., Krieg H. (2010). A consistent and accurate ab initio parametrization of density functional dispersion correction (DFT-D) for the 94 elements H-Pu. J. Chem. Phys..

[28] Vilela Oliveira D., Laun J., Peintinger M.F., Bredow T. (2019). BSSE-correction scheme for consistent gaussian basis sets of double—and triple-zeta valence with polarization quality for solid-state calculations. J. Comput. Chem..

[29] Kvitka N.G., Zorina S.S. (1969). Refinement of the structure of the spinel Al_2_MgO_4_. Sov. Phys.Crystallogr..

[30] Edlmayr V., Pohler M., Letofsky-Papst I., Mitterer C. (2013). Microstructure and thermal stability of corundum-type (Al_0.5_Cr_0.5_)_2_O_3_ solid solution coatings grown by cathodic arc evaporation. Thin Solid Films.

[31] Bondioli F., Ferrari A.M., Leonelli C., Manfredini T., Linati L. (2000). Reaction Mechanism in Alumina/Chromia (Al_2_O_3_–Cr_2_O_3_) Solid Solutions Obtained by Coprecipitation. J. Am. Ceram. Soc..

[32] Mahat A.M., Mastuli M.S., Kamarulzaman N. (2016). Influence of annealing temperature on the phase transformation of Al_2_O_3_. AIP Conf. Proc..

[33] Gagné O.C., Hawthorne F.C. (2015). Comprehensive derivation of bond-valence parameters for ion pairs involving oxygen. Acta Crystallogr. Sect. B Struct. Sci. Cryst. Eng. Mater..

[34] Bosi F., Andreozzi G.B. (2017). Chromium influence on Mg-Al intracrystalline exchange in spinels and geothermometric implications. Am. Mineral..

[35] Cámara F., Bindi L., Pagano A., Pagano R., Gain S.E.M., Griffin W.L. (2019). Dellagiustaite: A novel natural spinel containing V^2+^. Minerals.

[36] Hanson B., Jones J.H. (1998). The systematics of Cr^3+^ and Cr^2+^ partitioning between olivine and liquid in the presence of spinel. Am. Mineral..

[37] Liu C., Hystad G., Golden J.J., Hummer D.R., Downs R.T., Morrison S.M., Ralph J.P., Hazen R.M. (2017). Chromium mineral ecology. Am. Mineral..

