# Supplementary figures and images for: The Formation of Cr-Al Spinel under a Reductive Atmosphere
Source: Materials (Basel). 2021 Jun 10;14(12):3218. doi: 10.3390/ma14123218 (PMC8230446; doi:10.3390/ma14123218)

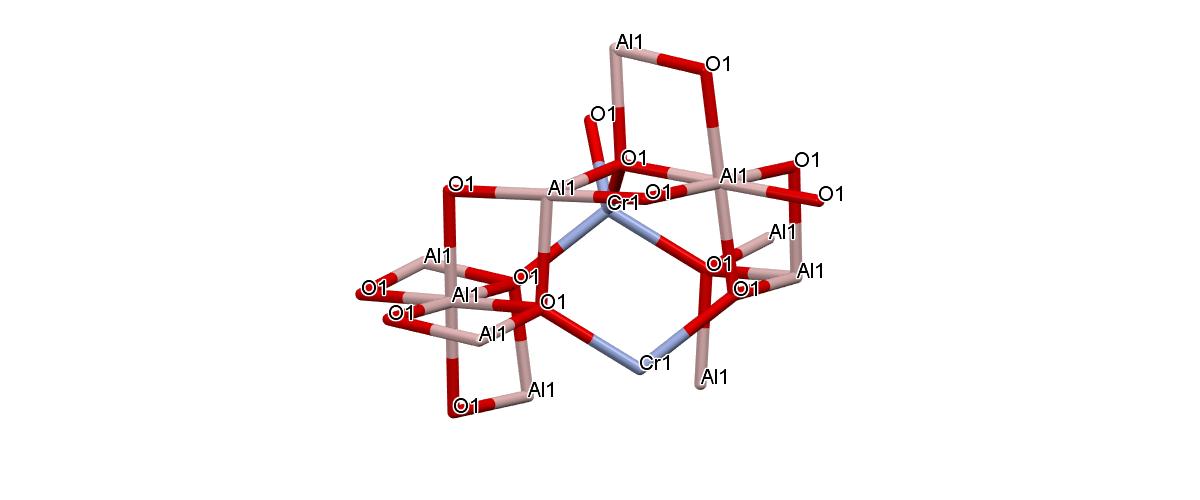

Supplement: Supplementary file 1 [file materials-14-03218-s001.zip › CrAl2O4.jpg]
